# Bioreactor mechanically guided 3D mesenchymal stem cell chondrogenesis using a biocompatible novel thermo-reversible methylcellulose-based hydrogel

**DOI:** 10.1038/srep45018

**Published:** 2017-03-23

**Authors:** A. Cochis, S. Grad, M. J. Stoddart, S. Farè, L. Altomare, B. Azzimonti, M. Alini, L. Rimondini

**Affiliations:** 1 Laboratory of Biomedical Materials, Department of Health Sciences, Università del Piemonte Orientale “UPO”, Novara, Italy; 2 Consorzio Interuniversitario Nazionale per la Scienza e Tecnologia dei Materiali (INSTM), Firenze, Italy; 3 AO Research Institute Davos, Davos Platz, Switzerland; 4 University Medical Center, Albert-Ludwigs University Freiburg, Germany; 5 Department of Chemistry, Materials and Chemical Engineering “G. Natta”, Politecnico di Milano, Milano, Italy; 6 Laboratory of Applied Microbiology, Department of Health Sciences, Università del Piemonte Orientale “UPO”, Novara, Italy

## Abstract

Autologous chondrocyte implantation for cartilage repair represents a challenge because strongly limited by chondrocytes’ poor expansion capacity *in vitro.* Mesenchymal stem cells (MSCs) can differentiate into chondrocytes, while mechanical loading has been proposed as alternative strategy to induce chondrogenesis excluding the use of exogenous factors. Moreover, MSC supporting material selection is fundamental to allow for an active interaction with cells. Here, we tested a novel thermo-reversible hydrogel composed of 8% w/v methylcellulose (MC) in a 0.05 M Na_2_SO_4_ solution. MC hydrogel was obtained by dispersion technique and its thermo-reversibility, mechanical properties, degradation and swelling were investigated, demonstrating a solution-gelation transition between 34 and 37 °C and a low bulk degradation (<20%) after 1 month. The lack of any hydrogel-derived immunoreaction was demonstrated *in vivo* by mice subcutaneous implantation. To induce *in vitro* chondrogenesis, MSCs were seeded into MC solution retained within a porous polyurethane (PU) matrix. PU-MC composites were subjected to a combination of compression and shear forces for 21 days in a custom made bioreactor. Mechanical stimulation led to a significant increase in chondrogenic gene expression, while histological analysis detected sulphated glycosaminoglycans and collagen II only in loaded specimens, confirming MC hydrogel suitability to support load induced MSCs chondrogenesis.

Effective treatment of cartilage defects represents a challenging problem, mainly due to the tissue’s limited intrinsic self-repair capacity. Currently, the only FDA-approved cell-based therapy for cartilage defects involves autologous chondrocyte implantation: chondrocytes harvested from low-contact areas are expanded *in vitro* and then re-injected directly into the damaged site^1^. This procedure has reported promising results in early clinical studies^1^; however, the entire process is restricted by limited expansion ability of chondrocytes *in vitro*, difficulty in maintaining the chondrocyte phenotype *in vitro*, and donor site morbidity^2^,^3^.

Accordingly, investigations on cellular therapies have therefore moved to progenitor cell populations such as bone marrow derived mesenchymal stem cells (BMSCs), which have the ability to differentiate into cartilage cells^4^. Clinically, autologous BMSCs have been used to repair articular cartilage defects by surgical transplantation of collagen-embedded BMSC constructs^5^,^6^,^7^ and by direct intra-articular injections of BMSCs^8^.

Besides the cell source choice, also mechanical stimuli are crucial in the development and maintenance of articular cartilage. In fact, different forces are tightly related to the cartilage microenvironment: hydrostatic pressure (HP), tension, compression and shear^9^. Thus, to mimic natural tissue microenvironment, different bioreactor systems have been developed to reproduce the physiological loading conditions and have been used to investigate cartilaginous tissue formation *in vitro*^9^,^10^. These studies have demonstrated that mechanical forces are able to shape the mesenchymal stem cell (MSC) fate when appropriately applied in *in vitro* models of cartilage regeneration. The concept behind this strategy is that bioreactors can not only be used to precondition implants before surgery, but they represent an effective tool to study the cellular response to mechanical stimulation under defined conditions^9^. To achieve this goal, some groups designed bioreactors able to impose compression^11^,^12^, while other studies reported about systems that can apply controlled mechanical shear by means of frequency and amplitude^13^. Shear stress can also be obtained by applying external flux as described by Chan *et al*.^14^, but this strategy seems to be in contrast to the natural environment, as cartilage is not subjected to an external active flow and except for the perichondrium does not receive nutrients from the sides^15^. Accordingly, the bioreactor used in this work was selected as able to provide mechanical stress by means of a moving and rolling ceramic ball (as schematized in Fig. 1) suitable to mimic compressive and shear forces approximating the kinematic motion of an articulating joint to determine whether applied forces affected stem cells fate.

From a materials science point of view, several biomaterial scaffolds have been used to study the effect of dynamic loading on chondrocytes and MSCs^9^,^10^,^16^,^17^,^18^. In fact, there is a strong relationship between the mechanical signals sensed by the cells and the material properties. For example, tailored synthetic crosslinked poly(ethylene glycol) (PEG) hydrogels have been used to study the interactions with chondrocytes both in compressive static and in dynamic culture systems^16^. Over short culture periods, dynamic loading of 15% strain at 1 Hz did not significantly affect the chondrocyte extracellular matrix gene expression (Type I and II collagen and aggrecan) compared to the static model^16^. However, chondrocytes cultured in PEG hydrogel enriched with RGD peptides, which can act as a binding site for chondrocytes, showed a general gene expression upregulation under mechanical loading compared to the static cultures^17^. Hence, the importance of the interaction/binding between the encapsulating matrix and the chondrocytes for conveying mechanical signals was demonstrated^17^,^18^. In particular, MSC survival and differentiation are strongly dependent on their adhesion to the substrate they have been seeded on. Therefore, the material features resulting in a biocompatible scaffold are very important. From this point of view, recent literature showed that a novel interesting class of materials, suitable for biomedical applications, consists of smart polymers. Smart polymers are able to significantly change one or more physico-chemical and/or mechanical properties when an external driving force such as pH or temperature change is applied^19^,^20^,^21^,^22^. In particular, among them, smart hydrogels experience rapid changes in their microstructure from a hydrophilic to a hydrophobic state triggered by small changes in the environment^21^. Interestingly, the changes are reversible; therefore, the polymer is capable of returning to its initial state as soon as the trigger is removed. For these properties, the interest in smart hydrogels is steadily gaining increasing momentum especially in the fields of cell and drug delivery. In particular, delivery systems based on these smart polymers can be adapted to closely target the altered physiological process of the diseased state ensuring optimum drug or cell release. Particularly, thermo-responsive smart hydrogels can present two distinct phases, and the passage from the collapsed to the swollen state corresponds to a physical/mechanical change of the hydrogel from a liquid phase (sol phase), in which the polymer is dissolved, to a gel phase. In fact, water becomes a poorer solvent with increasing temperature, and polymer–polymer interactions become dominant at higher temperatures, resulting in a gel^19^,^20^,^21^,^22^. Accordingly, in the gel phase, the hydration solvent is repulsed, since the interactions energy between the polymer chains are predominant over the solvent-polymer interactions. On the opposite, in the sol phase, the hydration solvent is absorbed, because the polymer chain-hydration solvent becomes predominant over the chain-chain interactions^20^. Therefore, in the gel phase, the thermo-responsive hydrogel finally tends to gel because of the polymer chain network reticulation.

Among the large class of suitable materials to manufacture hydrogels, methylcellulose (MC) based gels demonstrate a thermo-reversible behavior due to the presence of both hydrophilic (-OH) and hydrophobic (-OCH_3_) groups^23^,^24^,^25^,^26^,^27^. As MC hydrogels exhibit a sol-gel phase transition in response to a temperature change, these hydrogels can be used as injectable biodegradable hydrogels for cell delivery and tissue engineering applications.

In this work, the suitability of a novel thermo-reversible MC-derived hydrogel formulation was tested as a 3D injectable matrix for bioreactor guided BMSC chondrogenesis in combination with a porous polyurethane scaffold. The MC hydrogel composition was selected by rheological characterization in order to optimize the phase transition at 37 °C, representing the physiological body temperature. Afterwards, the selected MC hydrogel biocompatibility was evaluated *in vivo*. Finally, the MC hydrogel was used as matrix for BMSCs retained within a porous polyurethane scaffold; the interpenetrated PU-MC composite (i.e. MC hydrogel into the PU foam) was mechanically stimulated with a bioreactor able to simultaneously apply compression and shear forces. All *in vitro* chondrogenesis studies were performed in the absence of exogenous transforming growth factor β. After 21 days the composites were collected and chondrogenesis was evaluated by RT-PCR, biochemical, histochemical and immunofluorescent assays demonstrating that the mechanical stimulation was effective in determining chondrogenic stem cell fate.

## Results and Discussion

### Hydrogel mechanical characterization

The mechanical characterization data of the selected MC-hydrogel are reported in Fig. 2. As shown in panel A, the MC hydrogel exhibited reversible thermo-responsive properties. Phase transition was confirmed by the rheological characterization. In fact, temperature increase can promote variations in hydrogel mechanical properties (Fig. 2B and C). The physical gelation of MC hydrogels was visually observed using the inversion method already described in the literature^26^,^28^. The measurements showed that the gelation temperature of the selected MC hydrogel occurs between 34 and 37 °C, allowing us to handle cell constructs for some minutes without losing stability as previously shown^28^. Moreover, the storage shear modulus (G′) and the loss shear modulus (G″) obtained from the temperature sweep test confirmed the sol-gel transition. At low temperature (approximately in the range 5–10 °C), G′ was lower than G″ due to the viscous/liquid-like behavior of the MC solution, i.e. sol phase. Increasing the temperature, G′ first showed a decrease, reaching a minimum, then it rapidly increased for the sol/gel transition (i.e., gel point), as a soft elastic gel is formed. When MC hydrogel was heated, an increase in G′ could be observed, due to the change in the macromolecular rearrangement in the MC hydrogel structure. Conversely, immediately after cooling, the hydrogel showed a decrease in the mechanical parameters thus indicating the transition to the sol state. These properties are certainly related to the MC chemical structure and are in accordance to those shown by literature^21^,^23^. In fact, it is characterized by both hydrophobic and hydrophilic groups represented by methoxy (-CH_3_) and hydroxy groups (-OH), respectively. At low temperatures (<10 °C), hydrophilic interactions between -OH groups and solvent are predominant, so MC macromolecules are hydrated and the polymer structure is held together by simple entanglements. As the temperature increases, the hydrogel absorbs energy and gradually loses the hydration water. Polymer-polymer interactions take place between -CH_3_ groups, forming a gel-network structure.

In the heating and cooling tests (Fig. 2C), a hysteresis is observed. The hysteresis between the heating and cooling processes may be due to the existence of some associated aggregates or weak connections that have not completely disassociated. Differences may also be due to a different dynamic in forming and destruction of the chain to chain interactions related to the different dynamic in absorbing and releasing water. Another factor that may affect the destruction of the polymeric structure is the time the system has equilibrated at the highest temperature before cooling. In this work, the cooling process started immediately after the heating process. However, if the sample is allowed to equilibrate for some time before cooling starts, the time can have an effect on the hydrogel behaviour during the cooling process. Samples are likely to be more difficult to dissociate because of the more homogeneous network formed, compared to a freshly formed gel at the same temperature and a different trend may be recorded.

### *In vitro* gels degradation and swelling

The 8% w/v MC Na_2_SO_4_ hydrogel was incubated in various environments in order to investigate polymer degradation and swelling. Swelling data (Fig. 2D) showed that the hydrogel reached a maximum swollen volume of 170% in PBS and 210% in water and media after 1 day, presenting a significant volume increase compared to day 0 (p < 0.05). At the following time points, the swollen volume further increased to finally achieve values between 210% (PBS), 260% (media) and 350% (water) after 30 days; these values were always significantly different compared to the day 0 and comparable with results previously shown for similar hydrogels^25^. According to the results reported in Fig. 2E, the hydrogel reported a slight degradation after the first 24 hours with a range between 1 to 20% for water, PBS and media, while it was higher (about 35%) for the acidic environment (i.e., HCl). In the time lap of 7-15-21-30 days, the values reported after 24 hours for water, PBS and media did not show significant increases (p > 0.05), demonstrating a degradation profile levelling off in the range of 10–30%. The only exception was HCl (positive control) that reported a significant degradation (range of 35–75%) compared to the other solvents. Therefore, the MC hydrogel reported encouraging results as possible cell carrier even for a relatively long (30 days) application when incubated in culture media.

### *In vivo* immunoreactivity

The MC hydrogel *in vitro* cytocompatibility was previously reported^28^; therefore, in order to propose it as medical tool for cartilage regeneration, its *in vivo* biocompatibility was tested after 3 and 6 weeks implantation (as schematized in Fig. 3A). Mice implanted with hydrogel (Fig. 3B–E) did not develop any macroscopic inflammatory reaction such as fibrosis (Fig. 3B, H/E staining representative for 6 weeks implants) within the 6 weeks tested. Spleens collected from animals implanted with biomaterial were also comparable to controls, revealing the absence of macroscopic evidences of inflammatory processes and comparable weights (Fig. 3C). When the Stimulating Index (SI) was calculated at each time point, the hydrogel-coated sample score was always <1 confirming that no inflammatory reactions were raised toward the hydrogel. On the opposite, lymphocytes stimulated with the mitogen conA reacted quickly, always reporting SI scores >1 (Fig. 3D). At 3 and 6 weeks after hydrogel specimen implantation, a statistically significant difference was noted between hydrogel and conA groups. These findings suggest that the collected primary lymphocytes were competent but not reactive towards MC hydrogel, confirming the polymer biocompatibility. Finally, red and white blood cells count (Fig. 3E) did not revealed any significant differences between hydrogel-implanted mice and controls (p > 0.05), thus confirming SI index results. These data are in accordance to previous literature showing the lack of MC toxicity^20^,^21^,^22^,^23^,^24^,^25^,^26^ and thus its suitability as dedicated material for tissue engineering purposes. Moreover, according to *in vitro*^28^ and here presented *in vivo* results, it can be concluded that the use of Na_2_SO_4_ salt in combination with MC does not lead to any inflammatory reaction.

### PCR Analysis

Gene expression values of polyurethane-hydrogel-MSC composites (PU-MC composite) after 21 days of mechanical stimulation with the bioreactor are reported in Fig. 4. The PU macroporous scaffold was included in order to retain the gel during the unconfined bioreactor study and would not be implanted. The 21 day end point was selected in accordance to previous results obtained by applying mechanical loading with the same bioreactor^9^,^10^ and the MC hydrogel mechanical characterization where it showed to well tolerate a 28 days immersion in DMEM (even if in static conditions). In general, samples exposed to compression and shear showed a higher expression of the selected genes when compared with the unloaded controls. Therefore, the mechanical forces of compression and shear were effective in affecting the cell fate during the 21 days stimulation. This has previously been shown to be a specific effect as compression alone, which would induce mass transfer, does not induce chondrogenesis of human MSCs^10^. The induction of chondrogenesis under these conditions relies on the induction and activation of endogenous TGF-β^29^,^30^. MSC expansion was under standard conditions that utilized MSC qualified bovine serum. This leads to a population of cells that are unable to undergo spontaneous chondrogenesis in the absence of chondrogenic stimuli. The *in vitro* differentiation study utilized chondropermissive medium that included dexamethasone (DEX). DEX is a non-specific facilitator of differentiation that is present in chondrogenic, adipogenic, and osteogenic medium. Other than the addition of ITS+ a non-specific supplement that increases cell survival of non-adherent cells in serum free medium, and ascorbic acid that is required for collagen synthesis, no other stimulatory molecules were present. That is confirmed by the lack of a chondrogenic response under static conditions. Considering that the data are normalized to the day 0, a clear induction of COL 2 expression was noticed after the loading period (Fig. 4A). The stimulated specimens reported a 2.5 × 10^4^ fold increase in COL 2 expression compared to day 0 values, while only a 1.6 fold increase was observed in controls. This confirms previous results from mechanically loaded MSC-seeded fibrin-PU constructs^10^ and represents an important finding, indicating the higher chondrogenic differentiation of MSCs guided by the bioreactor induced compression and shear forces. Another significant result is related to the regulation of SOX 9 expression (A); SOX 9 represents an important transcription factor characteristic of cells that are committed to the chondrocytic phenotype. As observed for COL 2, SOX 9 expression was increased by mechanical loading (64 fold increase); conversely, the control samples expressed very low levels of SOX 9. These data further corroborate that the loaded MSCs were successfully guided towards chondrogenic differentiation. In contrast, collagen type I (COL 1) was only up-regulated about 10.1 times after mechanical loading, which represents a favorable result, as COL 1 is strongly related with osteogenesis (A). Therefore, the obtained data for collagen I expression suggest that the combination of compression with surface shear was effective in promoting the chondrogenic rather than the osteogenic phenotype of MSCs in the present PU-MC composite. Accordingly, the strongly increased COL 2: COL 1 ratio (C) suggest that the matrix may progressively be composed mainly of the cartilage specific COL 2. Collagen 10 (COL 10) expression, which is representative for the hypertrophic phenotype, was also up-regulated by the mechanical load (A). Enhanced levels of hypertrophy markers related to the use of mechanical bioreactors have previously been observed^10^. It is therefore possible that the forces applied in this study were able to induce cell differentiation towards chondrogenesis but not sufficient to completely prevent further maturation towards hypertrophy. Nevertheless, the high COL 2: COL 10 ratio clearly demonstrates that the mechanical stimuli were able to delay the hypertrophic differentiation process (D).

This is an important finding because the lack of a continuous mechanical stimulation can lead to an increase in hypertrophy even if chondrogenesis in general is correctly induced^14^. Finally, aggrecan (ACAN) was found to be clearly overexpressed in loaded specimens (A) as well the ratio ACAN: COL 10 was significant (E) confirming the induction of the typical cartilage-like matrix genes. In conclusion, PCR analysis demonstrates that the mechanical stimulation was able to induce chondrogenesis in MSCs embedded in the MC hydrogel matrix.

### ALP activity

As a further confirmation of the loading induced chondrogenesis, ALP was used to determine any osteogenic-like differentiation. Results (Fig. 4B) revealed a very small amount of mineralized tissue that was not significant by comparing loaded and control specimens (p > 0.05). Finally, also ALP staining did not report any evident accumulation of mineralized matrix within control (not shown) and loaded PU-MC composites pores (B, upper panel). These findings correlate with the PCR results showing a low COL 1 expression; moreover, the strong influence provided by the presented bioreactor (at the same operating conditions) towards cartilage-like lineage differentiation is comparable to previous works when a porous PU scaffold was filled with fibrin glue^9^,^10^.

### Biochemical analysis

Biochemical evaluations are reported in Fig. 5. The number of cells within loaded and control specimens resulted as comparable, as DNA amount did not reported any significant difference at D21 (A, p > 0.05). Thus, even if a dedicated viability assay is lacking, it can be speculated that cells were similarly viable within the loaded and control PU-MC composites. Similarly, when GAG amount inside scaffold was evaluated, no significant differences were noticed (B, p > 0.05, lower panel), even if total GAG amount was superior for loaded specimens. This finding was more evident when safranin-O staining was applied to sections obtained by tested specimens (B, upper panel). Images showed an increased GAG accumulation in the loaded samples that was evident when compared to controls. The difference between GAG quantification and safranin-O is due to the uneven nature of mechanically induced chondrogenesis, with increased differentiation observed in the upper areas of the scaffold. Overall GAG/DNA quantification is an average that includes the non-responding cells, while the safranin-O staining provides localized information. The amount of glycosaminoglycans (GAGs) released into the medium was determined every 3 days, while the scaffold GAG content was measured at the end of the loading period (21 days). Values were normalized to the DNA content of the respective samples (C-D). In general, the data revealed a progressive increase in GAGs in the stimulated samples. This was particularly noticeable when looking at the GAG release into the medium (C); the loaded samples’ values steadily increased during the 21 loading days with significant data in comparison with controls after 18 days stimulation (p < 0.05, indicated by the asterisk). On the opposite, the GAGs accumulated in the medium of the control samples showed only a slight increase over the observation time. These data were also confirmed when the total amount of GAG accumulated in scaffold and medium was considered; the difference between loaded and control samples was statistically different (p < 0.05). The production of GAGs is an important phenotypic parameter indicating chondrogenic differentiation. Once MSCs acquire a chondrogenic phenotype, the challenge is to prevent them from becoming hypertrophic. Amongst others, this step is associated with a decrease in GAG secretion^9^. Looking at the time course of GAG secretion in the medium, values continue to increase up to day 21. Thus, it is possible to speculate that the loaded cells, even if COL 10 expression was positive, were not adopting a hypertrophic phenotype. The fact that GAGs were mostly recovered in the medium than in the scaffold is a common phenomenon when using a porous scaffold for cartilage repair^31^,^32^; in fact, a known limitation in using porous scaffolds is that they fail to accumulate most of the extracellular matrix proteins synthesized by the cells. From this point of view, it must be taken into account that protein exchange will be strongly influenced by mechanical deformation that also increases fluid exchange through the scaffold. This could explain increases in media GAG content of loaded scaffolds. However, total GAG synthesis (scaffold and medium) was significantly higher under loaded conditions, thus demonstrating an increased chondrogenic differentiation compared to static conditions. Thus, although cells are metabolically active (as confirmed by PCR findings and increased GAG synthesis), they seem to lack the appropriate environment or proper stimuli to produce a structured extracellular matrix within the timeframe of this experiment^9^. Moreover, by using fibrin glue as cells matrix in a newly developing construct, the bulk of the newly synthesized GAG was released into the culture medium as the pericellular matrix was not sufficiently developed to retain the newly produced GAG^32^,^33^,^34^. Therefore, the MC hydrogel seems to act similarly to fibrin according to obtained and previous results. According to most recent literature, polymer networks rich in water such as hydrogels are the most popular option for cartilage regeneration because up to 80% of articular cartilage wet weight consists of water^35^. Moreover, for cartilage repair, the use of injectable hydrogels is of special interest because they are compatible with arthroscopic methods. Hydrogels can be obtained using natural or synthetic polymers with different benefits and disadvantages. Natural polymers include alginate, agarose, and silk^36^; the unique composition of these polymers makes them unrecognizable to human enzymes, allowing slow degradation and more time for the body to initiate and support regeneration^36^. Moreover, these hydrogels have been shown *in vitro* to provide suitable environments to maintain the phenotype of encapsulated chondrocytes^36^. However, they are normally poor in terms of mechanical properties and lack natural attachment sites for cells and inherent bioactivity to trigger synthesis of extracellular matrix^37^. The here presented MC-based hydrogel shows a highly tuneable ability to customize mechanical properties in function of specific needs^28^ resulting in a superior suitability as cell carrier in comparison to the other natural derived hydrogels. Moreover, MC-based hydrogels have been shown in literature to be able to support mature chondrocytes proliferation and typical GAG and COL II rich matrix synthesis^38^,^39^,^40^ providing the necessary microenvironment to drive and sustain chondrogenesis.

If synthetic hydrogels for cartilage repair such as polyglycolic acid (PGA) and polylactic acid (PLA) are considered, their major drawback consists in the poor ability to promote cells adhesion and proliferation^41^. As a consequence, they normally must be doped with active biomolecules (such as dexamethasone and transforming growth factor beta); thus, once implanted in patients, materials efficacy is dramatically related to biomolecules retention and lifespan, lacking a justification for clinical transition. As prior debated, MC-hydrogel here presented was not enriched with any molecules, thus missing the major limitation of synthetic hydrogels.

Finally, it should be noted that even macroporous scaffolds do lead to an increase in pericellular matrix within 3 weeks of culture. As the pericellular matrix matures a greater fraction of the newly synthesized matrix is retained and the disadvantage of the macroporous scaffold is reduced. During the process of cartilage regeneration in patients, which is over months or years, the initial low level of deposition is unlikely to play a major role.

### Histology

Loaded and control samples were investigated and compared for COL 1, COL 2 and COL 10 deposition by means of immunofluorescence (IF) as further confirmation of PCR data. All markers staining are reported in Fig. 6. IF revealed the presence of COL 1 in small amounts (A, lower panel, in green) in loaded samples, while no signals were detected in the control samples, thus confirming the PCR data. The IF confirmed the presence of COL 2 within almost all the examined scaffold pores of the loaded samples (B, lower panel, in green). The control samples did not show any positive staining for collagen II, as previously indicated by the PCR analysis (B, upper panel). Finally, COL 10 was detected in loaded specimens (C, lower panel, in green) but in a lower amount in comparison with COL 2, thus confirming that cells were not adopting a hypertrophic phenotype due to the continuous mechanical stimulation provided by daily bioreactor operating. These data are in accordance to literature as compressive stress combined with shear flow allows a higher MSC commitment^11^,^33^,^34^,^42^. Hence, IF staining confirmed the PCR data, providing further important indication of the successful bioreactor-guided chondrogenesis. Nonetheless, according to the PCR analysis, it was expected to detect a higher amount of collagen II in the scaffolds. A possible explanation of this discordance is that COL 2 synthesis at the protein level in MSCs is known to be slow^9^. Second, as the matrix produced is relatively immature a large proportion is likely to have been released into the medium. Finally, it must be taken into account that collagen deposition resulted as randomly strayed after loading as showed by Kock *et al*.^42^. Natural cartilage presents a fountain-like collagen structure^15^ that normally requires months to develop. Due to the short nature of *in vitro* studies, collagen remodeling is normally not achieved.

## Conclusions

The aim of the work was to evaluate the suitability of the MC hydrogel as 3D matrix to support the mechanical induction of chondrogenesis in human MSCs. Due to the thermo-responsive behavior and its biocompatibility, MC hydrogel represents a good candidate to act as vehicle for cell delivery. As practice example, when it was used as cell carrier in combination with a PU scaffold, a positive chondrogenic response was achieved. In fact, the mechanically stimulated MSCs successfully expressed chondrogenic genes and the GAG quantification confirmed the higher differentiation route of the MSCs. Histological analysis confirmed the retention of the cells within the PU scaffold pores and the presence of a surrounding matrix of collagen and proteoglycan, confirming the suitability of MC-based hydrogel as cell carrier for MSC based cartilage repair.

## Methods

### Methylcellulose-derived (MC) thermo-reversible hydrogel preparation

A 8% w/v methylcellulose (MC, Methocel A4M, η = 4000 mPa×s for a 2% w/v aqueous solution at 20 °C, Sigma Aldrich, Milan, Italy) hydrogel was prepared with a dispersion technique in a 0.05 M Na_2_SO_4_ solution, as previously described by Altomare *et al*.^28^. Hydrogel was prepared using filter-sterilized solution (0.22 μm pore size filters, VWR International, Milan, Italy) and UV light sterilized powder.

### Mechanical characterization

Rheological characterization was performed with a rotational rheometer (AR-1500ex, TA Instruments, New Castle, USA), using a flat plate geometry (diameter = 2 cm, working gap = 1 mm) and a custom-made isolation chamber in polymethyl methacrylate (Plasting srl, Milan, Italy) to prevent hydrogel dehydration. Tests were performed using six replicates. Dynamic Complex viscosity (η*), storage shear modulus (G′) and viscous shear modulus (G″) were acquired over the 5–50 °C temperature range, with a temperature rate of 5 °C/min. The oscillation frequency during the temperature ramp was maintained at 1 Hz. Thermo-reversibility characteristic of the MC hydrogel was studied with a first run increasing the temperature from 4 up to 40 °C and a second run decreasing the temperature down to 4 °C (T ramp = 10 °C/min, oscillation frequency = 1 Hz).

### Degradation and Swelling

Swelling and degradation tests were performed by adding 300 μl of MC polymer solution (T = 4 °C) in 24-well plates, where samples were allowed to equilibrate at 37 °C for 2 hours. Then, 1 ml of Dulbecco’s Modified Eagle’s Medium (DMEM, Sigma Aldrich, Milan, Italy) supplemented with 10% fetal bovine serum (FBS, Sigma Aldrich, Milan, Italy) or phosphate buffered saline (PBS, Sigma Aldrich, Milan, Italy), or 0.5 N HCl (Carlo Erba, Milan, Italy), was added to each well containing the MC hydrogel (*n* = 6 for each gel/media combination). After 1, 7, 15, 21 and 30 days, the media was removed and the weight of hydrogels was evaluated. Afterwards, MC samples were air-dried and the resulting polymer mass was weighted. Swelling and degradation were evaluated in function of time and calculated as follow^43^:




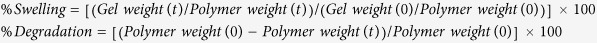




### *In vivo* immunoreactivity evaluation

Animal surgical procedures were approved by the local ethics committee and the relevant research institute (Università del Piemonte Orientale, Novara, Italy); compliance with the NIH Guide for Care and Use of Laboratory Animals was guaranteed. Surgery procedures were performed under 3% isoflurane general anesthesia. One centimetre diameter specimens of the MC hydrogel were subcutaneously implanted into the dorsal skin of 6–8 weeks old wild type mice (C57BL/6JOlaHsd, Harlan Laboratories, Udine, Italy). After 3 and 6 weeks, cellular immune response was determined by using a spleen primary lymphocyte proliferation assay as described by VandeVorde *et al*.^44^. Cells were suspended (cell density = 2.5 × 10^6^ cells/mL) in Alpha Minimal Essential Medium (α-MEM, Sigma Aldrich, Milan, Italy) supplemented with 10% FBS (Sigma Aldrich, Milan, Italy), 1% antibiotics (penicillin-streptomycin, Sigma Aldrich, Milan, Italy). Then, 500 μl of cell suspension were spotted into the wells of a 24 well plate previously coated with 100 μl of hydrogel or PBS as negative control. Positive controls consisted of cells stimulated with the mitogen ConA (5 mg/mL) to confirm lymphocytes competence. After 48 hours cell viability was evaluated by MTT assay (Sigma, 3 mg/ml in PBS); cellular responses were expressed as Stimulation Index (SI), by comparing mean optical density of cells cultured in the presence of hydrogel (ODh) and ConA (ODcA) and mean OD of cells without hydrogel, used as control (ODC); SI was calculated from the following formulas^44^ considering scores > 1 as responsive:




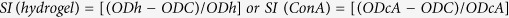




Blood specimens were collected and cell numbers evaluated by counter machine (Cell Countess, IBM, New York, USA). Spleens were collected and weighted immediately after sacrifice.

### Isolation and expansion of human bone marrow-derived mesenchymal stem cells

The human MSCs were obtained from fresh bone-marrow aspirates of patients undergoing surgery at the University hospital of Freiburg. All subjects gave informed consent to participate to the study in accordance with the institutional ethical committee of the Albert-Ludwigs University of Freiburg, Germany (EK-Freiburg, approval nr. 135/14) and in fully compliance with the Declaration of Helsinki. Bone marrow stromal cells were isolated from 4 donors (2 Male/2 Female; 19–49 years old; mean: 38y, detailed in Supplementary Table 1) by standard density gradient procedure (Histopaque-1077, Sigma Aldrich, Milan, Italy) and selection by plastic adherence. Bone marrow-derived mesenchymal stromal cells (MSCs) were cultured in α-MEM, 10% human MSC qualified foetal bovine serum (FBS-HyClone, Thermo Fisher Scientific, Waltham, USA) with 5 ng/mL fibroblast growth factor 2 (FGF2, Fitzgerald Industries, Acton, USA). At 70–80% confluence, cells were harvested and used for the experiment at passage 2–3. Each experiment was performed separately in quadruplicate for each donor and the data collected for statistics.

### MC- hydrogel-polyurethane composite culture of MSCs

Cylindrical (8 mm diameter × 4 mm height) porous elastic polyurethane (PU) scaffolds (pore size of 90–300 μm) were prepared as described elsewhere^45^. MSCs were suspended in the MC hydrogel before seeding into the PU scaffolds. 3 × 10^6^ cells were suspended in 160 μl hydrogel in the sol state, and then filled into each PU scaffold by compressing and releasing it several times until all the hydrogel was absorbed. Constructs were then incubated for 2 hours at 37 °C in incubator, to allow MC hydrogel gel-sol transition before adding a serum-free chondropermissive medium (DMEM, with 4.5 g/L glucose and 2.2 g/L NaHCO_3_, non-essential amino acids, containing 11.5 mg/L L-proline, 50 μg/mL ascorbic acid 2-phosphate sesquimagnesium salt hydrate, ITS+1 (10 μg/mL insulin from bovine pancreas, 5.5 μg/mL human transferrin (substantially iron-free), 5 ng/mL sodium selenite, 0.5 mg/mL bovine serum albumin and 4.7 μg/mL linoleic acid;, 10^−7 ^M dexamethasone, 100 U/mL penicillin + 100 μg/mL streptomycin, all from Sigma Aldrich, Milan, Italy). After 2 days pre-culture in 12-well plates, PU-MC composites were transferred in polyether ether ketone (PEEK) holders mounted onto the bioreactor system^46^. Medium was changed every 3 days and collected for further analysis.

### Bioreactor

**Figure 1:**
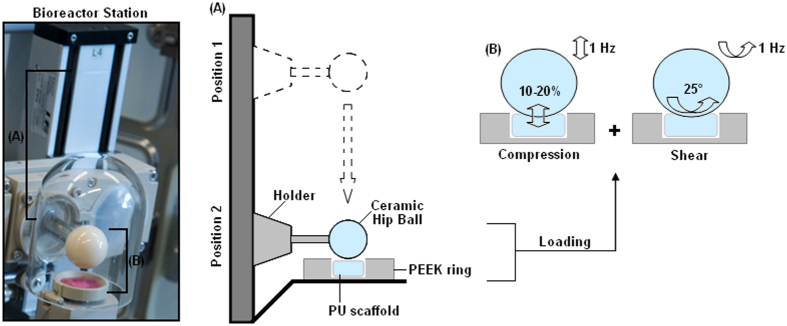
Schematic representation of bioreactor operation. The single-station bioreactor image (left) shows the mobile holder carrying the ceramic hip ball (marked by **A**) used to mechanically stress the polyurethane scaffold (PU) held in the polyether ether ketone (PEEK) ring (marked by **B**). Accordingly, as schematized for **A** and **B** in the cartoon on the right, mechanical loading is provided by the ceramic hip ball that is held by a mobile metallic holder; by software remote control, the holder can move from stand-by Position 1 to operative Position 2 (**A**) to reach the PU that is maintained within the PEEK ring. Here, the ball compresses the PU scaffold dynamically at 1 Hz resulting in strain amplitude of 10–20% of its height (**B**, left panel). Simultaneously, the ball oscillates ±25° at 1 Hz to provide mechanical shear stress (**B**, right panel).

**Figure 2:**
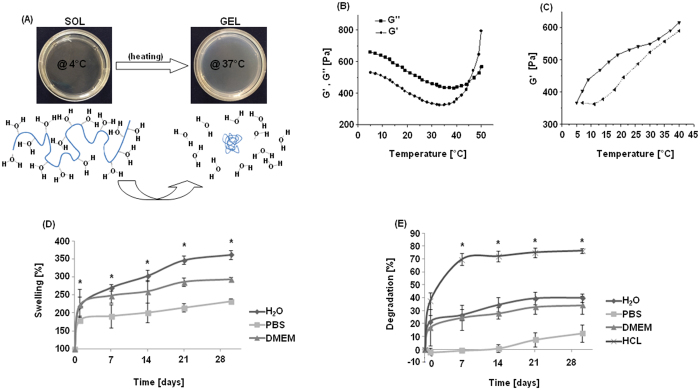
Representative pictures of MC hydrogel sol and gel phases (**A**, upper panel) and schematic representation of physical transition (**A**, lower panel, MC in blue). Shear storage modulus (G′, round dots) and shear loss modulus (G″, square dots) versus temperature (**B**). Thermo-reversible behavior of the MC hydrogel increasing (straight line) and decreasing (dot line) the temperature (**C**). Percent swelling of MC hydrogel versus incubation time (**D**); at each time point a statistical difference between day and day (1-7-15-21-30) was detected, while the asterisk (*) represents a significant difference between water and media (p < 0.05). Gel degradation rates are reported in (**E**): asterisk (*) indicates a significant difference (p < 0.05) between acid (HCl, positive control) and other incubation environments.

**Figure 3:**
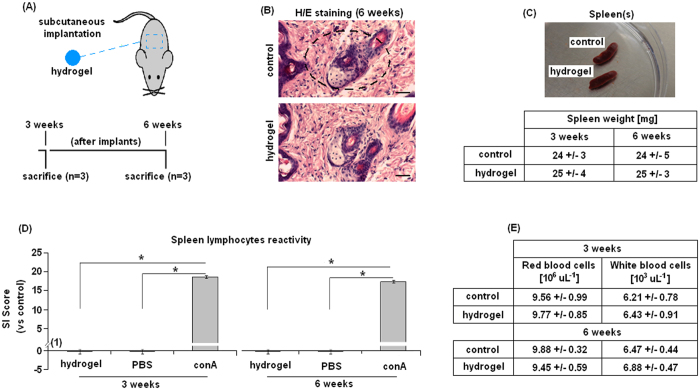
*In vivo* immunological reaction. Hydrogel cylindrical samples were implanted subcutaneously in the mice dorsal skin and collected 3 and 6 weeks after implantation (**A**). Six weeks implants histological analysis by hematoxylin/eosin (H/E) staining did not report any differences between tissues implanted with hydrogel (implant site indicated by square) and controls (**B**); moreover, at each time point spleens appeared comparable with controls (**C**, representative for the 6 weeks) with comparable weight (detailed in the Table). The Stimulation Index (SI) score (**D**) revealed that no immune reaction was caused by the hydrogel implants, whereas lymphocytes correctly reacted towards conA; red and white blood cells count (**E**) confirmed SI assay results. Bars represent means and standard deviations; H/E bar scale = 50 μm.

**Figure 4:**
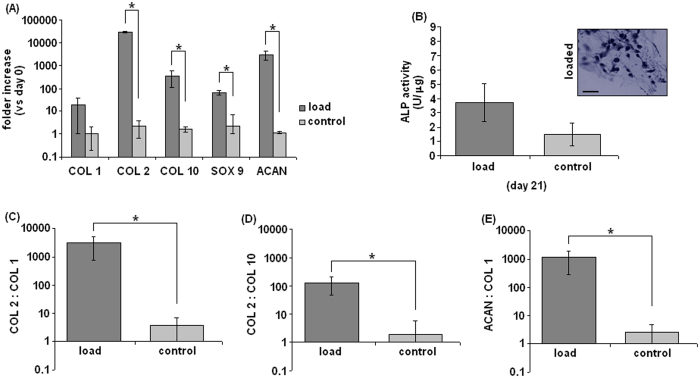
Relative mRNA expression of human mesenchymal stem cells after 21 days of stimulation normalized to day 0 (**A**). Loaded samples showed a clear up-regulation of chondrogenic markers collagen II (COL 2) and SOX 9; also the hypertrophy marker COL 10 was induced; however, the ratio COL 2: COL 10 was significant (**D**, p < 0.05, indicated by the asterisk). Collagen I (COL 1) was also expressed markedly in the loaded specimens (**A**) but the ratio COL 2: COL 1 was still significant (**C**, p < 0.05, indicated by the asterisk) suggesting a higher up-regulation of the COL 2 and thus confirming MSC chondrogenesis. Aggrecan (ACAN) was found to be clearly overexpressed in loaded specimens confirming the presence of the typical cartilage-like matrix genes (**A**). Moreover, the ratio ACAN: COL 1 was again significant (**E**, p < 0.05, indicated by the asterisk). Finally, as further confirmation that stem cell fate was strongly influenced towards chondrogenesis rather than osteogenesis, ALP activity in the scaffold (**B**) was low and difficult to be detected (**B**, upper panel representative for loaded scaffolds) and no significant differences were noticed between loaded and control specimens (p > 0.05). Bars represent means and standard deviations; all data were normalized to day 0 values. The loaded samples values always resulted as statistically different compared with unloaded (control) samples (p < 0.05, indicated by the asterisk). Bar scale = 100 μm (magnification 40x).

**Figure 5:**
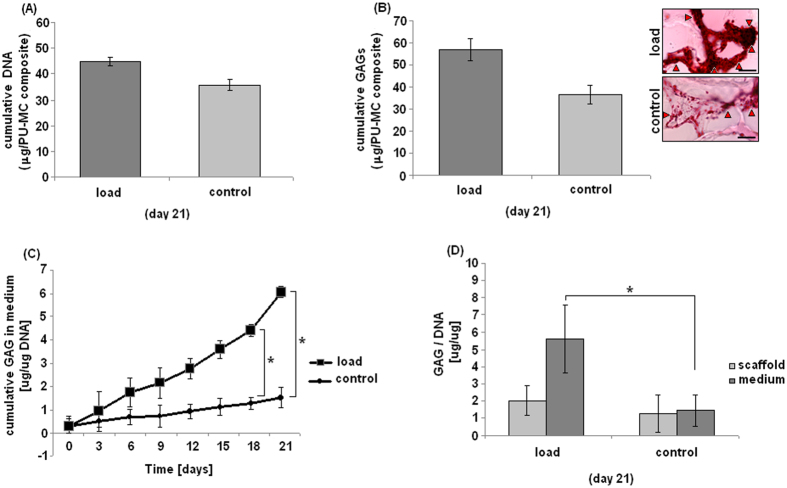
GAG evaluation. After 21 days loading the number of cells in the loaded and control specimens was comparable as they showed a similar DNA amount (**A**, p > 0.05). The GAG/scaffold amount was higher for the loaded ones, but not in a statistical manner in comparison with controls (**B**, p > 0.05, lower panel). Differences were also appreciable by safranin-O staining as shown in (**B**), upper panel. Conversely, accumulated GAG released in the medium over 3 weeks of culture normalized to the DNA content of respective samples (**C**) showed significant results over the loading period of 18 days (p < 0.05, indicated by the asterisk). After 21 days the scaffold GAG amount was determined and the GAG amount of medium and scaffold compared between load and control (**D**). The total GAG amount was significantly different between loaded and control samples (p < 0.05, indicated by the asterisk). Bar scale = 100 μm, (magnification 40x).

**Figure 6:**
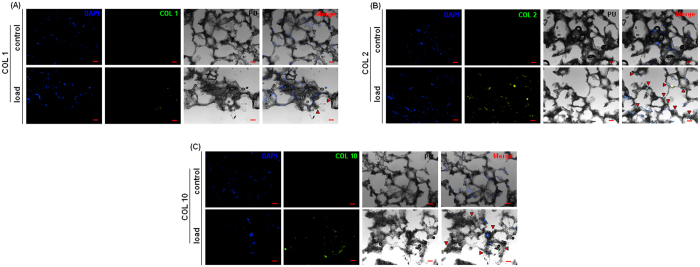
Collagen I (COL 1), collagen II (COL 2) and collagen X (COL 10) staining. Control samples did not show COL 1 presence in the matrix (**A**, upper panel), while a small amount was found within the pores containing the matrix of loaded cells (**A**, lower panel, indicated by red arrows). Control samples did not exhibit COL 2 immunoreactivity (**B**, upper panel), while the loaded ones clearly showed the COL 2 presence in almost all the stained scaffold pores (**B**, lower panel, highlighted by red arrows). Finally, COL 10 signal was found only in the loaded specimens (**C**, lower panel, indicated by red arrows) but not in the control ones (**C**, upper panel). For all markers, merging DAPI and collagen fluorescent signals with the bright field image of the polyurethane (PU) scaffold confirmed the intra-pore localization (Merge). Bar scale = 100 μm.

Mechanical conditioning of the cell-scaffold PU-MC composites was performed by using a pin-on-ball bioreactor system (as schematized in Fig. 1). Briefly, a 32 mm diameter ceramic ball was pressed onto the scaffold^10^. Interface shear motion was generated by oscillation of the ball about an axis perpendicular to the scaffold axis. Superimposed compressive strain was applied along the cylindrical axis of the composites. Samples were exposed to unconfined dynamic compression at 1 Hz with 0.4 mm sinusoidal strain, superimposed on a 0.4 mm static offset strain, resulting in strain amplitude of 10–20% of the scaffold height at the centre of the construct. Simultaneously, samples were also exposed to ball oscillation of ±25° at 1 Hz^10^,^45^,^46^,^47^. Mechanical load was applied during 1 hour/day for 21 consecutive days. PU-MC composites, not loaded in the bioreactor, were used as controls. Experiments were carried out in quadruplicate for each donor for both loaded and not loaded sample configurations.

### Analysis

After 3 weeks of culture and 21 loading cycles, PU-MC composites were vertically cut in two halves; for each donor and group (loaded and unloaded control), 3 scaffold halves were processed for biochemical analysis, 3 for gene expression analysis and 2 for histological and immunohistochemical analysis.

### Gene expression

PU-MC composites used for gene expression analysis were homogenized in 1 mL TRI reagent and 5 μL Polyacryl Carrier (both Molecular Research Center, Cincinnati, USA) per scaffold, using a Tissue-Lyser (Retsch & Co, Haan, Germany) and centrifuged (Eppendorf, Basel, Switzerland) at 4 °C for 10 min at 12000 *g*. RNA isolation was carried out according to the protocol of the manufacturer. RNA was reverse transcribed with TaqMan reverse transcription kit (all from Applied Biosystems, Foster City, USA) using random hexamers. For real time PCR TaqMan Gene Expression Assays or custom designed primer-probe sets (from Microsynth AG, Balgach, Switzerland) were used in a GeneAmp 7500 Real Time PCR System (Applied Biosystems, Foster City, USA). The endogenous control gene was 18 S rRNA. Chondrogenic (collagen type-II (COL 2), SRY (sex determining region Y) – box 9 (SOX 9)), aggrecan (ACAN), osteogenic/fibroblastic (collagen type-I (COL 1)) and hypertrophic markers (collagen type-X (COL 10)) were analyzed. The primers and probes used are listed in Table 1. Gene expression was analyzed according to the ^ΔΔ^Ct method, with expression levels normalized to the corresponding day 0 sample (i.e. day of cell seeding into the scaffolds) of each donor.

### Alkaline Phosphatase (ALP) activity

ALP activity was determined by a biochemical colorimetric assay using an alkaline phosphatase kit (Gene Tex, Irvine, USA) after 21 days loading cycle following manufacturer’s instructions. Results were normalized towards protein amounts obtained by a BCA protein assay kit (Pierce Biotechnology, Rockford, USA).

### Biochemical analysis

PU-MC composites were digested with 0.5 mg/mL proteinase K at 56 °C overnight and used for DNA and glycosaminoglycan (GAG) measurement. DNA concentrations were determined with the Hoechst method using calf DNA as a standard. Fluorescence intensity was measured with an HTS 7000 Perkin Elmer Bio Assay Reader (Perkin Elmer, Milan, Italy). The amount of glycosaminoglycan (GAG) was determined by the dimethylmethylene blue dye method, using bovine chondroitin sulphate as standard. The total GAG content of the culture media, collected every 3 days, was also measured to assess the release of matrix molecules from the sample into the media. Absorbance was measured with a Victor3 Perkin Elmer 1420 multilabel counter (Perkin Elmer, Milan, Italy). GAG values were normalized to the DNA content.

### Histochemistry and immunofluorescence

PU-MC composites were fixed in 70% methanol at 37 °C to prevent hydrogel solid-gel phase transition and incubated in 5% D^(+)^ sucrose (Sigma Aldrich, Milan, Italy) solution in PBS for 12 h at 37 °C before embedding them in Jung tissue freezing compound and cryosectioning at 10 μm (Microm HM560 CryoStar, Thermo Scientific, Waltham, USA)^10^. The presence of GAGs was investigated using safranin-O (Sigma Aldrich, Milan, Italy). The deposition of collagen type I, type II and type X was determined by an immunofluorescence staining. After enzyme pre-treatment (0.5 U/mL Hyaluronidase), sections were incubated with antibodies against collagen I (COL 1, 1:150, rabbit polyclonal, ab34710 from Abcam, Cambridge, UK), collagen II (COL 2, 1:50, rabbit polyclonal, ab34712 from Abcam, Cambridge, UK) and collagen X (COL 10, 1:50, rabbit polyclonal, ab58632 from Abcam, Cambridge, UK). Sections were incubated overnight at 4 °C, washed 3 times with PBS and co-stained using an appropriate secondary antibody (Alexa-Fluor conjugated anti-rabbit, 1:500 in PBS, AP132JA4 from Sigma Aldrich, Milan, Italy). Samples were visually investigated by a fluorescence microscope (Leica DM5500 B, Leica Microsystems, Basel, Switzerland).

### Statistical analysis

Statistical analysis was performed using the software package SPSS (Version 20, SPSS Inc, IBM, New York, USA). Student’s *t-test* was used to analyze difference. The significance level was defined at *p* < 0.05.

## Additional Information

**How to cite this article:** Cochis, A. *et al*. Bioreactor mechanically guided 3D mesenchymal stem cell chondrogenesis using a biocompatible novel thermo-reversible methylcellulose-based hydrogel. *Sci. Rep.* **7**, 45018; doi: 10.1038/srep45018 (2017).

**Publisher's note:** Springer Nature remains neutral with regard to jurisdictional claims in published maps and institutional affiliations.

## Supplementary Material

Supplementary Information

## Figures and Tables

**Table 1:** Primers and probes summary.

Gene	Abbreviation	Forward (5′–3′)	Riverse (5′–3′)	Probe (5′FAM/3′TAMRA)
Collagen type I	COL 1	CCC TGG AAA GAA TGG AGA TGA T	ACT GAA ACC TCT GTG TCC CTT CA	CGG GCA ATC CTC GAG CAC CCT
Collagen type II	COL 2	GGC AAT AGC AGG TTC ACG TAC A	GAT AAC AGT CTT GCC CCA CTT ACC	CCT GAA GGA TGG CTG CAC GAA ACA TAC
Collagen type X	COL 10	ACG CTG AAC GAT ACC AAA TG	TGC TAT ACC TTT ACT CTT TAT GGT GTA	ACT ACC CAA CAC CAA GAC ACA GTT CTT CAT TCC
Aggrecan	ACAN	AGT CCT CAA GCC TCC TGT ACT CA	CGG GAA GTG GCG GTA ACA	CCG GAA TGG AAA CGT GAA TCA GAA TCA ACT
**Gene**	**Abbreviation**	**Applied Biosystems Serial number**		
SYR box-9	SOX 9	Hs_00165814_m1		
ribosomal 18 S	18 S	4310893E		
