# Supplementary material for: Bioreactor mechanically guided 3D mesenchymal stem cell chondrogenesis using a biocompatible novel thermo-reversible methylcellulose-based hydrogel
Source: Sci Rep. 2017 Mar 23;7:45018. doi: 10.1038/srep45018 (PMC5362895; doi:10.1038/srep45018)
Supplement: Supplementary Information [file srep45018-s1.doc]

**Title: Bioreactor mechanically guided 3D mesenchymal stem cell chondrogenesis using a biocompatible novel thermo-reversible methylcellulose-based hydrogel**

*Authors and Affiliations:*

Cochis A^1,5^, Grad S^2^, Stoddart MJ^2,6^, Farè S^3,5^, Altomare L^3,5^, Azzimonti B^4,5^, Alini M^2^ and Rimondini L^1,5*^

1. *Laboratory of Biomedical Materials, Department of Health Sciences, Università del Piemonte Orientale “UPO”, Novara, Italy.*
2. *AO Research Institute Davos, Davos Platz, Switzerland.*
3. *Department of Chemistry, Materials and Chemical Engineering “G. Natta”, Politecnico di Milano, Milano, Italy.*
4. *Laboratory of Applied Microbiology, Department of Health Sciences, Università del Piemonte Orientale “UPO”, Novara, Italy.*
5. *Consorzio Interuniversitario Nazionale per la Scienza e Tecnologia dei Materiali (INSTM), Firenze, Italy.*
6. *University Medical Center, Albert-Ludwigs University Freiburg, Germany*

********Corresponding Author*:

Prof. Lia Rimondini

Laboratory of Biomedical Materials

Department of Health Sciences, Università del Piemonte Orientale “UPO”

Via Solaroli 17, 28100 Novara (NO), Italy

E-mail: [lia.rimondini@med.uniupo.it](mailto:lia.rimondini@med.uniupo.it)

Tel: +39 0321 660673

Fax: +39 0321 620421

| **MSCs Donor #** | **Age** | **Sex** | **Nationality** |
| --- | --- | --- | --- |
| 1 | 19 | M | Germany |
| 2 | 42 | M | Germany |
| 3 | 49 | F | Germany |
| 4 | 44 | F | Germany |

**Supplementary Table 1**. Bone marrow-derived mesenchymal stem cells (MSCs) Donors.
